# Retroperitoneal Fibrosis: Beware of Lymphoma

**DOI:** 10.7759/cureus.17587

**Published:** 2021-08-31

**Authors:** Mohamed Ouchani, Houda Bachir, Siham Hamaz, Habiba Alaoui, Khalid Serraj

**Affiliations:** 1 Internal Medicine, Immunohematology and Cellular Therapy Laboratory, Faculty of Medicine and Pharmacy, Mohammed First University, Oujda, MAR; 2 Infectious Disease, Immunohematology and Cellular Therapy Laboratory, Faculty of Medicine and Pharmacy, Mohammed First University, Oujda, MAR

**Keywords:** r-chop, retroperitoneal fibrosis, follicular lymphoma, biopsy of rpf, corticosteroid therapy, chemotherapy

## Abstract

Retroperitoneal fibrosis is a rare disease manifesting as chronic soft tissue fibrosis in the retroperitoneum, with potential anatomic and/or functional compromise of adjacent organs. It can be primary (idiopathic) or secondary to other conditions such as cancers, radiotherapy, surgery, traumatisms, infections, autoimmune disorders, or drugs. We report herein a 54-year-old patient with symptomatic retroperitoneal fibrosis leading to bilateral hydronephrosis and renal failure, in whom, after a complex diagnostic workup and protracted clinical course, a follicular lymphoma in the retroperitoneal was identified. The patient was treated with rituximab and cyclophosphamide, doxorubicin, vincristine, and prednisone (CHOP) chemotherapy protocol, with a resolution of hydronephrosis and lower back pain. We include a thorough literature review on etiopathogenesis, diagnosis, therapy, and prognosis of retroperitoneal fibrosis. A meticulous search for malignancy is necessary for this rare condition that, if positive, may have significant therapeutic and prognostic implications.

## Introduction

Retroperitoneal fibrosis (RPF) is a rare condition characterized by engorgement of the retroperitoneal structures by aberrant fibro-inflammatory tissue. Many underlying diseases, including infections, autoimmune diseases, certain neoplasia, asbestosis, certain drugs, radiotherapy, or previous major surgery, have been associated with the occurrence of RPF. According to the literature [1], these different causes account for approximately one-third of RPF cases [2]. The diagnosis of primary or “idiopathic” RPF is based on exclusion, thus raising a crucial issue of requiring formal histological evidence to retain the diagnosis of RPF in the face of compatible imaging.

We report a rare case of follicular lymphoma mimicking retroperitoneal fibrosis after the one recently reported by Yoichi Hoshino’s team from Keiaido Hospital in Japan [3].

## Case presentation

The patient is a 54-year-old carpenter who had alcohol and tobacco habits presented with asthenia with low back pain in March 2018. A thoracic-abdominal-pelvic computed tomography scan on April 16, 2018, revealed a left uretero-hydronephrosis secondary to retroperitoneal fibrosis, which led to the installation of a JJ stent. The evolution was marked by the relative improvement of symptoms during the first few months, but then the situation deteriorated, motivating the introduction of a treatment based on tamoxifen (40 mg/day) for five months in association with corticotherapy at a dose of 40 mg/day with digression until reaching 5 mg/day.

 A computed tomography (CT) scan on April 15, 2019, showed persistence of fibro-inflammatory tissue encasing the abdominal aorta and the iliac arteries, with permeable ureters. The patient was then lost to follow-up until March 2020, when his clinical condition worsened, marked by the intensification of lumbar pain, edema of the right thigh, and the onset of renal failure with a creatinine level of 24 mg/l and clearance of 31.9 ml/min/1.73 m^2^, justifying his transfer to our department. A CT scan on March 3, 2020, showing a large retroperitoneal tissue infiltrate extending from the lumbar region opposite L1 to the pre-sacral region, including the aorta, abdominal vena cava, iliac vessels, superior mesenteric artery, and renal vascular vessels as well as the ureters bilaterally, with caliceal dilatation and infiltration of the retroperitoneal fat. Left inguinal adenopathy measuring 30*20 mm was also discovered.

A positron emission tomography (PET-CT) scan was not performed for this patient. The hemoglobin was 11.2 g/dl, C-reactive protein (CRP) was 5.75 mg/l, erythrocyte sedimentation rate (ESR) was 30 mm/h, beta-2-globulin was 7.1 mg/l, and low-density lipoprotein (LDH) was 202 UI/l. The etiological tests included antinuclear antibody, antineutrophil cytoplasmic antibody (ANCA), QuantiFERON-TB Gold test, prostate-specific antigen (PSA) were within the normal range, with an IgG4 level of 0.076 g/l, and HIV serologies for viral hepatitis B and C were negative. The electrophoresis of proteins (EPP) showed hypoalbuminuria and hypogammaglobinemia.

**Figure 1:**
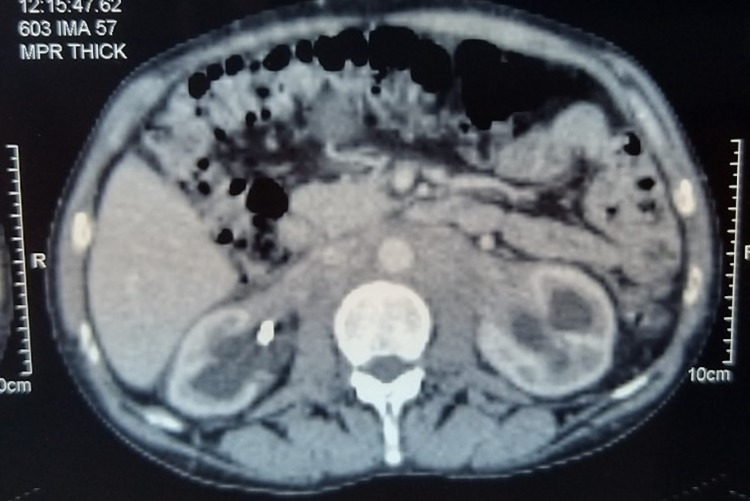
Abdominal CT scan, after contrast product injection, showing retroperitoneal fibrosis with a perivascular fibrous sheath and bilateral hydronephrosis.

In June 2020, the patient developed a deep vein thrombosis of the right thigh following the change of the JJ stents. A CT scan on June 5, 2020, showed a significant increase in posterior mediastinal and retroperitoneal tissue infiltration encompassing the vascular structures and ureters bilaterally in addition to a large bilateral pleural effusion (Figure 1).

Histopathological and immunohistochemical examination of the inguinal lymph node biopsy was in favor of a grade I follicular lymphoma. the pleural biopsy was without signs of malignancy. The bone marrow biopsy showed a reactive marrow appearance without tumor infiltration.

**Figure 2:**
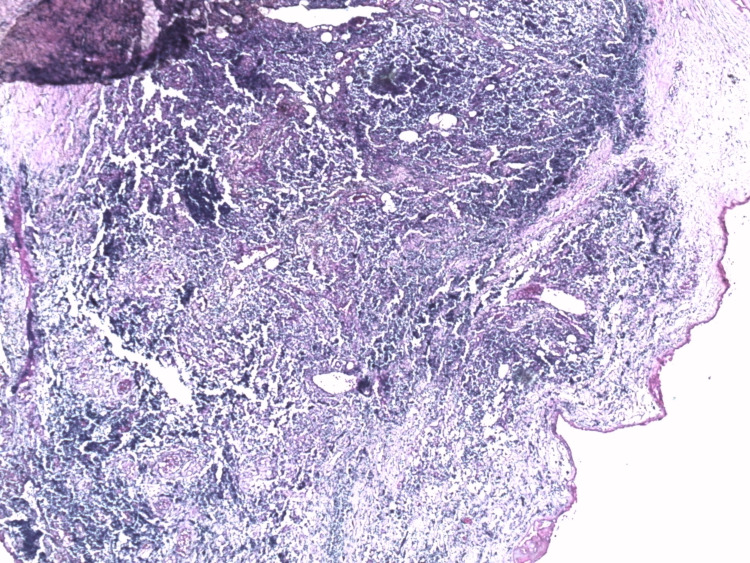
An atypical lymphoid infiltrate suggestive of a lymphoproliferative disorder (x 50).

**Figure 3:**
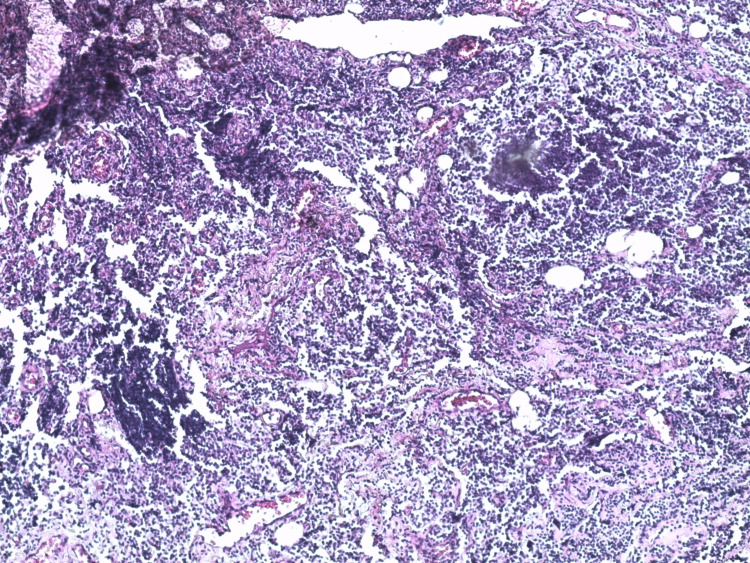
An atypical lymphoid infiltrate suggestive of a lymphoproliferative disorder (x 100).

**Figure 4:**
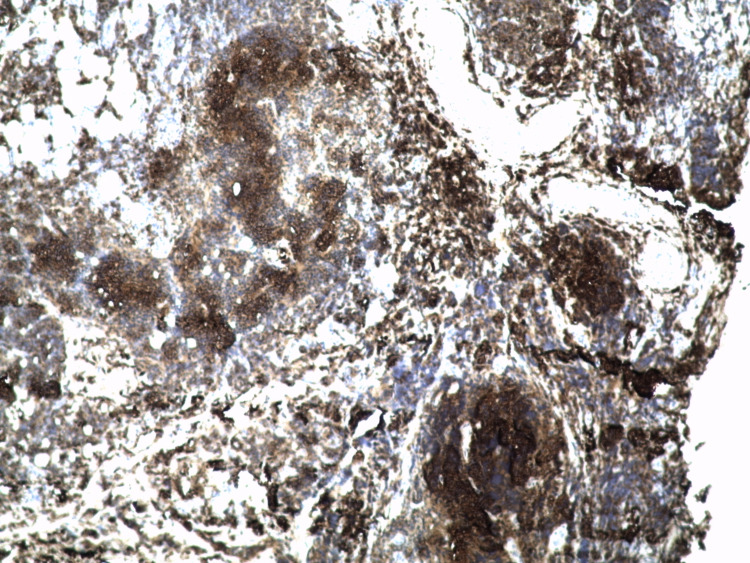
Intensive expression of CD31 (+) (x 100). CD: cluster of differentiation

**Figure 5:**
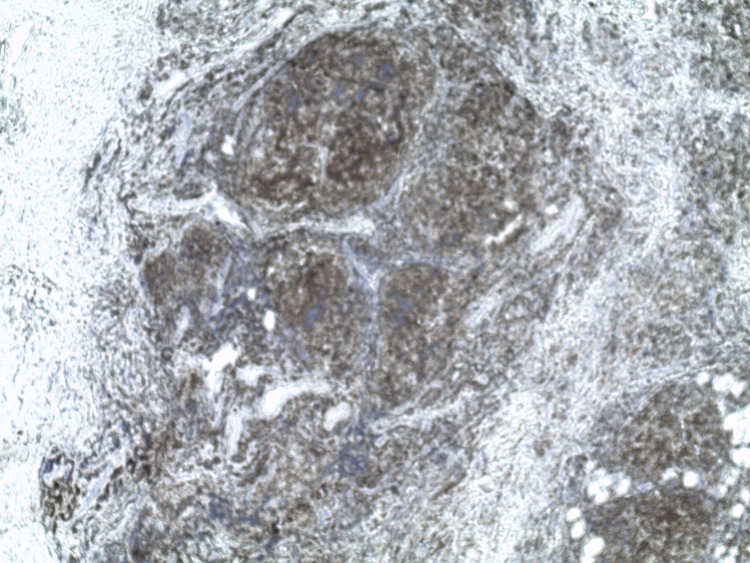
Proliferation of Bcl2 (+) on nodules (x 50). Bcl: B-cell lymphoma

**Figure 6:**
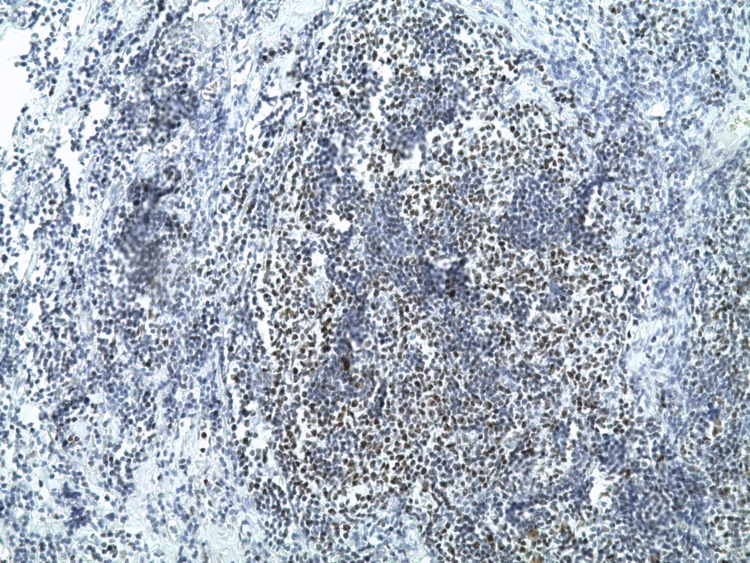
Proliferation of Bcl6 (+) on nodules (x 200). Bcl: B-cell lymphoma

**Figure 7:**
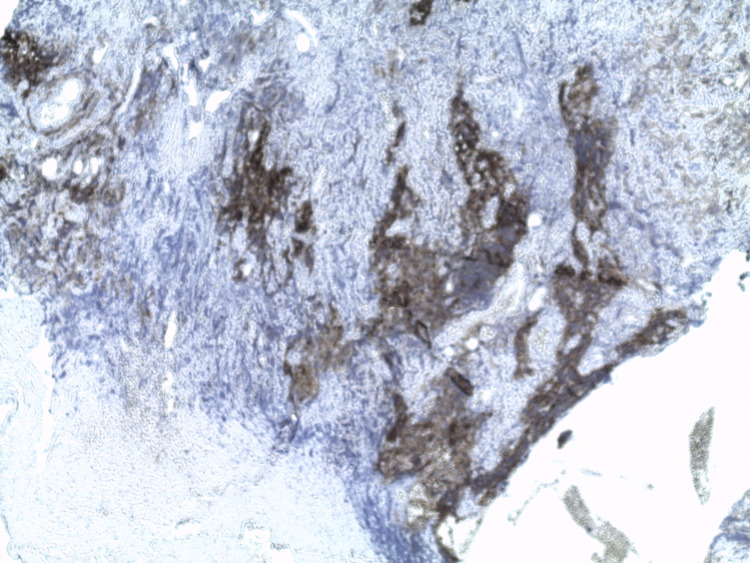
A disorganized follicular dendric cells (CD21 {-}) (x 50). CD: cluster of differentiation

**Figure 8:**
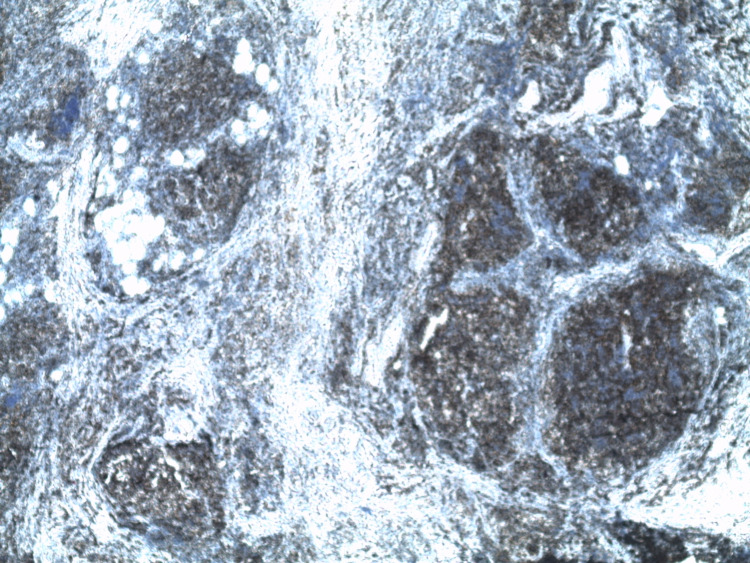
Proliferation of CD10 (+) on nodules (x 50). CD: cluster of differentiation

The decision was to put the patient on curative anticoagulation and 60 mg of prednisone for four weeks with progressive digression and subsequent biopsy of the retroperitoneal fibrous tissue whose immunohistochemical data are in favor of a follicular non-Hodgkin's lymphoma of grade I/WHO 2017 with cells expressing cluster of differentiation (CD)20, B-cell lymphoma (Bcl) 2, CD10, Bcl 6, T cells in inter-follicular tissue expressing CD5 (Figures 2-8).

Given the scannographic progression of retroperitoneal tissue infiltration and the pathological data, the decision was to put the patient on 04 cures of rituximab, cyclophosphamide, doxorubicin, vincristine, and prednisone (R-CHOP) protocol with a good clinical and radiological evolution.

## Discussion

RPF is a rare disease. Its first description dates back to Albarran in 1905, but it only became an anatomical-clinical entity with Ormond in 1948 [4]. It is characterized by the progressive transformation of the retroperitoneal adipose tissue into a fibrous mass, which encloses the abdominal aorta, inferior vena cava, and ureters. This mass is usually limited between the renal pedicle above and the sacral promontory below. However, extensions to the large peritoneal cavity, mediastinum, and pelvis, in particular, have been reported. It mainly affects men aged between 40 years and 60 years, with a mean age of 56 years [5,6].

The physiopathology of this condition remains unknown, and several hypotheses have been proposed, the most widely accepted being an autoimmune origin. The physiopathology of this condition remains unknown, and several hypotheses have been proposed, the most widely accepted being an autoimmune origin. In addition to idiopathic forms, which account for 70% of all RPF [1], there are secondary fibroses: to medication (methysergide, pergolesi, beta-blockers, hydralazine, amphetamines, haloperidol, reserpine, etc.), to retroperitoneal aggression (trauma, radiotherapy, surgery, etc.), or to inflammatory aneurysm of the abdominal aorta [7,8]. RPF can also be due to a malignant process in 10% of cases (colon, rectum, stomach, breast, prostate, lymphoma), and only a biopsy can differentiate between benign and malignant RPF [1]

RPF is sometimes associated and contemporaneous with inflammatory diseases, such as sclerosing cholangitis, sclerosing pancreatitis, Riedel's thyroiditis, retrobulbar pseudotumor, and mediastinal fibrosis [2].

The pathophysiology of RPF is still poorly understood, but the autoimmune hypothesis has been validated by several studies. The IgG-mediated inflammatory process and the presence of CD4 T cells, B cells, and macrophages within the fibrosis suggest an underlying lymphoproliferative process [9]. Furthermore, the immuno-hematological abnormalities found in RPF place the disease within the spectrum of lymphoid dyscrasias [7,9].

The clinical presentation is non-specific, highly variable, and often related to the mechanical effect of the RPF on the surrounding structures: abdominal and lumbar pain (80%), renal colic, edema of the lower limbs, deep vein thrombosis, testicular pain, hydrocele, and intermittent claudication of the lower limbs. General symptoms (fatigue, anorexia, weight loss, etc.) are frequent, reflecting the inflammatory nature of the disease [10]. The initial clinical presentation does not differentiate idiopathic RPF from secondary forms. It is therefore important to systematically look for predisposing factors as well as signs suggestive of an underlying autoimmune disease, particularly IgG4 disease [2].

Biologically, there is often an inflammatory syndrome (increased CRP and ESR, hyperleukocytosis), normocytic normochromic anemia, or thrombocytosis. In 70% of cases, there may also be polyclonal hypergammaglobulinemia. False positives for autoantibodies (antinuclear antibodies, rheumatoid factor, ANCA), advanced renal failure, disturbance of liver tests, false positives for tumor markers, and normal urine are also common [11].

CT and MRI are the examinations of choice for radiological diagnosis. They allow the fibrosis plaque to be demonstrated, specification of its morphology, and the identification of its location and spread to neighboring structures [12]. They may also reveal other disorders of IgG4 disease, such as autoimmune pancreatitis and chronic pericarditis with mediastinal fibrosis. The typical appearance is a well-limited but irregular mass of periaortic tissue extending from the level of the renal arteries to the iliac vessels, progressing into the retroperitoneum and enveloping the ureters and inferior vena cava. The fibroinflammatory tissue may extend down to the pelvis above the renal hilum, or in rare cases, it may invade the duodenum, renal hilum, or kidney [13].

Although CT and MRI are the best tools for the diagnosis of RPF, revealing the more or less extensive perivascular tissue zone that takes contrast after injection, such contrast taking testifies to the early and active character of the fibrosis plaque [12]. Two elements must be considered. First, there may be a genuine RPF even though imaging does not show a retroperitoneal plaque, in which case only the consequences on the urinary tract are visible. In addition, there is no formal difference in imaging between RPF of benign origin and RPF of malignant origin [14].

As an alternative, 18F-fluorodeoxyglucose (18F-FDG) PET has a very high sensitivity, superior to that of CT or MRI, with several advantages [13,15]. It enables exhaustive assessment of the inflammatory process in idiopathic forms and evaluates the intensity of the inflammatory process in the active phase [13]. Moreover, it can be used to detect the progression of the disease. It has also been proposed for identifying the best site for biopsy, which can be performed surgically or under radiological control [12-15].

In 10% of cases, RPF is the result of a malignant process of solid cancer (colon, rectum, stomach, breast, prostate) or hematological malignancies, such as lymphoma [1]. Only a few such cases have been reported in the literature. This makes it essential to perform an RPF biopsy before accepting the idiopathic origin and before starting corticosteroid therapy, which can delay the diagnosis, as was the case with our patient.

The principles guiding treatment are to relieve symptoms, preserve renal function by removing the obstruction caused by fibrosis, prevent invasion of other organs by stopping the progression of the fibrotic process, avoid recurrence, and exclude malignancy. A review of the literature over the last 20 years shows that the treatment of RPF is still empirical [11].

There is no publication with a sufficient level of evidence to define an optimal surgical strategy or pharmacological treatment with corticosteroids, which was introduced in 1958 by Ross and Tinckler [16]. Corticosteroids are effective in the early “inflammatory” stage of the disease but not in the late “sclerotic” stage [17].

RPF relapse when they are stopped. Other drugs have been used such as immunosuppressants, including tamoxifen, mycophénolate mofétil, and recently colchicine. Surgery consists of creating an emergency urinary diversion (percutaneous nephrostomy, ureteral catheters, JJ stents) or performing ureterolysis [11], accompanied by various procedures for distancing the ureters from the fibrous process, with a risk of recurrence or ureteral stenosis of 12-50% [11]. Etiological treatment remains crucial and depends on the found cause.

The prognosis of patients with idiopathic RPF is often better compared to that of patients with RPF secondary to malignancy. When malignancy is found to be in the advanced stages, there are limited options available [6]. However, when a secondary cause (e.g., lymphoma) is diagnosed, as in the case of our patient, appropriate and timely therapy can result in significant improvement of symptoms and a reduction in complications from RPF.

## Conclusions

This case shows an atypical presentation of follicular non-Hodgkin's lymphoma in the form of retroperitoneal fibrosis. Although this presentation is rare, any unexplained RPF should be investigated for malignancy, and biopsy with anatomopathological examination must be performed due to the therapeutic and prognostic implications.
